# The Investigation on Mechanical Performances of High-Strength Steel Reinforced Concrete Composite Short Columns under Axial Load

**DOI:** 10.3390/ma15010329

**Published:** 2022-01-03

**Authors:** Jun Wang, Xinran Wang, Yuxin Duan, Yu Su, Xinyu Yi

**Affiliations:** School of Civil Engineering, Northeast Forestry University, Harbin 150000, China; yuxind666@163.com (Y.D.); vincentsheart@outlook.com (Y.S.); yixinyu19981030@163.com (X.Y.)

**Keywords:** high-strength steel, steel-reinforced concrete composite short column, bearing capacity, ductility, ABAQUS simulation

## Abstract

At present, the existing standards (AISC360-16, EN1994-1-1:2004, and JGJ138-2016) lack relevant provisions for steel-reinforced concrete (SRC) composite columns with high-strength steel. To investigate the axial compressive mechanical performance of short high-strength steel-reinforced concrete (HSSRC) columns, the axial load test was conducted on 12 short composite columns with high-strength steel and ordinary steel. The influences of steel strength, steel ratio, and the section form of steel on the failure modes, bearing capacity, and ductility of the specimens were studied. Afterward, the experimental data were compared with the existing calculation results. The results show: compared with the specimens with Q235 steel, the bearing capacity of the specimens with Q460 steel increases by 7.8–15.3%, the bearing capacity of the specimens with Q690 steel increases by 13.2–24.1%, but the ductility coefficient increases by 15.2–202.4%; with the increase of steel ratio, the bearing capacity and ductility of specimens are significantly improved. A change of the steel cross-section could influence the ductility of SRC columns more than their bearing capacity. Moreover, the calculation results show that present standards could not predict the bearing capacity of HSSRC columns. Therefore, a modified method for determining the effective strength of steel equipped in HSSRC columns was proposed. The results of the ABAQUS simulation also showed that the addition of steel fibers could significantly improve the bearing capacity of Q690 HSSRC columns. The research results provide a reference for engineering practices.

## 1. Introduction

With the rapid development of high-rise and long-span buildings, ordinary construction materials do not meet the requirements of modern buildings. Since the 1990s, high-strength materials have increasingly been applied because their use in high-rise buildings can reduce the section size of structural elements and can reduce the use of building materials while saving building space. Therefore, it is more economical and environmentally friendly. In this background, high-strength and high-performance materials have gotten significant attention [1,2,3,4]. High-strength steel has excellent mechanical performance and can improve the mechanical performance of the structure. For the mechanical performance of high-strength steel, the calculation method based on density functional theory is proposed from the atomic level, which effectively reveals the mechanism of metal strength and provides guidance for industrial production [5,6]. In recent years, the production of high-strength steel has been increasing, and the welding conditions matching high-strength steel are also perfected, which lays a strong foundation for the extensive application of high-strength steel.

Scholars have carried out extensive research on the application of high-strength steel in the construction field. Some of them had applied high-strength steel bars to ordinary steel-concrete structures and found that high-strength steel bars can significantly improve the bearing capacity and ductility of reinforced concrete columns [7,8]. Compared with high-strength steel bars, high-strength steel tubes can better restrain concrete [9,10,11]. Restricted concrete can delay or even avoid local buckling of steel tubes so that the strength of steel tubes can be fully utilized [12,13,14]. Compared with the normal reinforced concrete columns, steel-reinforced concrete (SRC) columns have better structural performance, which can effectively reduce the cross-sectional size of structural columns and obtain larger building space. Compared with concrete-filled steel tubular columns, SRC columns can make full use of the compressive performances of high-strength steel, and poured concrete outside the steel can also avoid steel corrosion and improve the fire resistance of components [15,16]. Therefore, SRC structures have been widely used in key parts of important buildings [17,18,19]. The application of high-strength steel to SRC columns aims to improve the bearing capacity of the member without increasing or reducing the sectional area of the vertical member [20,21,22].

However, as shown in Table 1, during the study, scholars [23,24,25,26,27,28,29,30,31,32,33,34,35,36,37,38,39] also found that when high-strength steel is applied to building structures, the existing standards cannot accurately predict the mechanical performance of this type of specimen. Besides, design approaches from standards, including AISC360-16 [40], EN1994-1-1: 2004 [41], and JGJ138-2016 [42], are based on the related research results of ordinary steel; therefore, the scope of application needs to be verified.

**Figure 1:**
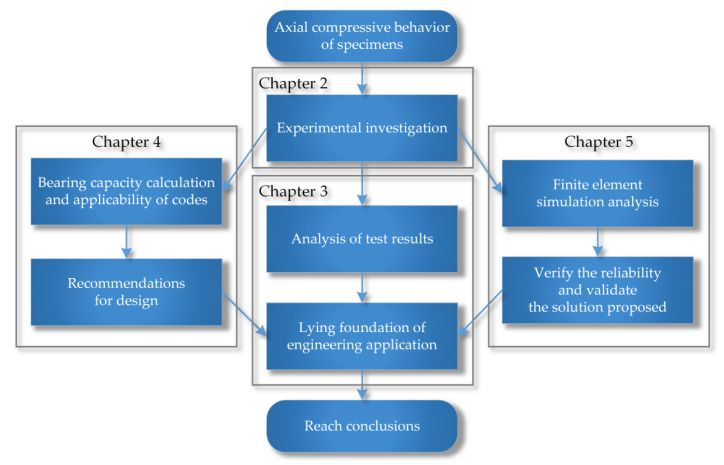
Flowchart of main steps.

To investigate the influence of steel strength, steel ratio, and the section form of steel on the axial compressive bearing capacity of HSSRC composite short columns, 12 specimens equipped with Q235, Q460, and Q690 were tested. The main steps of this research are shown in Figure 1. The experimental results were compared with the calculation results from existing related codes to verify the applicability of the calculation approaches, and design suggestions were proposed. Finally, finite element models were established.

## 2. Experimental Investigation

**Figure 2:**
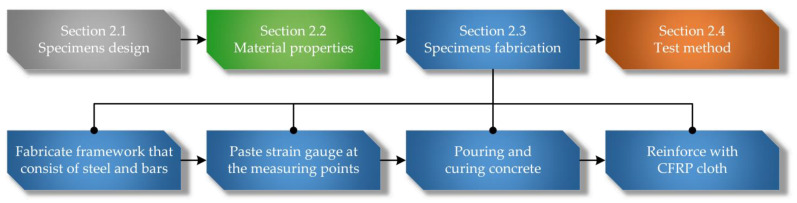
Flowchart of the experimental investigation.

This section contains four parts, and the main steps are shown in Figure 2.

### 2.1. Test Specimens

**Figure 3:**
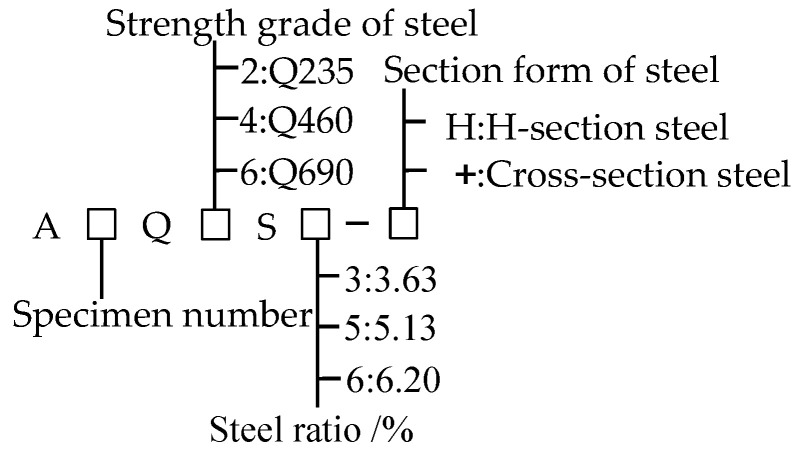
Labeling rule of specimens.

**Figure 4:**
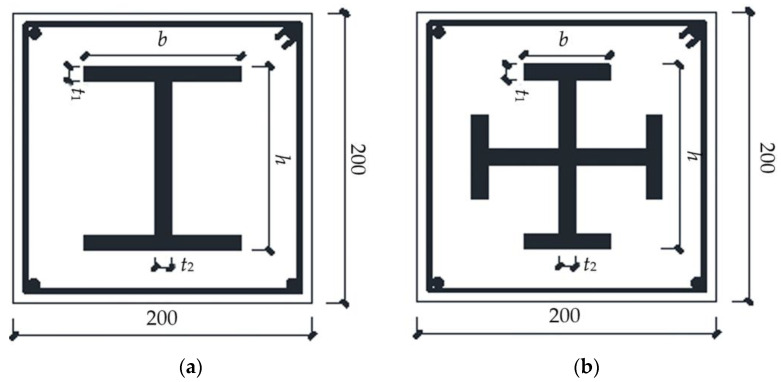
Cross-section and reinforcement of specimens (dimensions are presented in (mm)). (**a**) Specimens equipped with H-section steel. (**b**) Specimens equipped with cross-section steel.

A total of eight high-strength welded steel-concrete composite short column specimens and four ordinary welded steel concrete comparison specimens were designed for the experiment. The change parameters contain the strength grade of the section steel, the steel ratio, and the section form of the section steel. Figure 3 shows the details; the explanation of the number of the specimens is shown in Table 2; the section size and reinforcement of the specimen are shown in Figure 4. The height of the specimens was 600 mm. Considering that high-strength concrete has strong brittleness and poor crack resistance, C50 pea gravel concrete was used in this test.

For Simplicity, *A_i_* (*i* = 1~12) is used in some parts of the article to represent the number of specimens.

### 2.2. Material Properties

According to the relevant standard “Metallic Materials Tensile Test” (GB/T 228.1-2010) [43], the mechanical properties of the section steel and the steel bars were tested, as shown in Table 3.

According to the relevant standard “Standard for Test Methods of Concrete Structures” (GB/T 50152-2012) [44], the mechanical properties of the concrete were tested. Nine concrete cubes (150 mm × 150 mm × 150 mm) were maintained under the same conditions as the specimens in 28 days, and the cubic compressive strength of the concrete test block was 55.8 MPa.

### 2.3. Process of Specimen Manufacture

**Figure 5:**
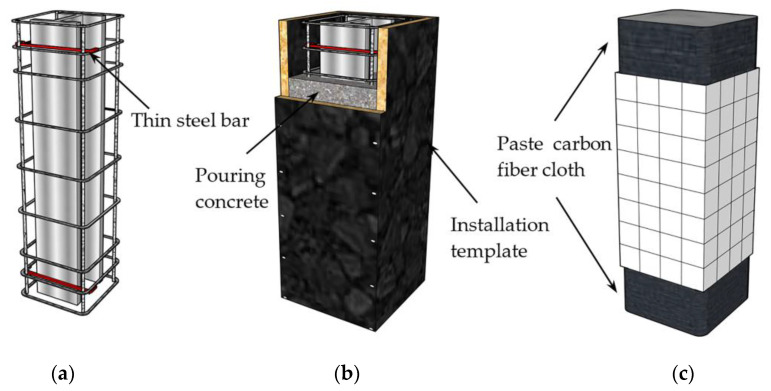
Manufacturing process of specimens. (**a**) Fix steel, (**b**) install formwork and pour, (**c**) paste carbon fiber cloth.

The production processes of the specimens are shown in Figure 5. The section of steel was formed by the full welding of three steel plates. As shown in Figure 5a, before pouring the specimens, the section of steel was fixed to the reinforcing steel frame by thin steel bars at the upper and lower ends. Further, paste strain gauges were placed at locations to be measured. As shown in Figure 5b, we installed the formwork and poured the concrete into layers that were fully vibrated during the pouring process. As shown in Figure 5c, after the curing was completed, the top surface of the specimen was polished flat. In order to prevent local pressure damage to the specimen, the upper and lower ends were reinforced with carbon fiber cloth within a range of 200 mm.

### 2.4. Test Setup and Procedure

**Figure 6:**
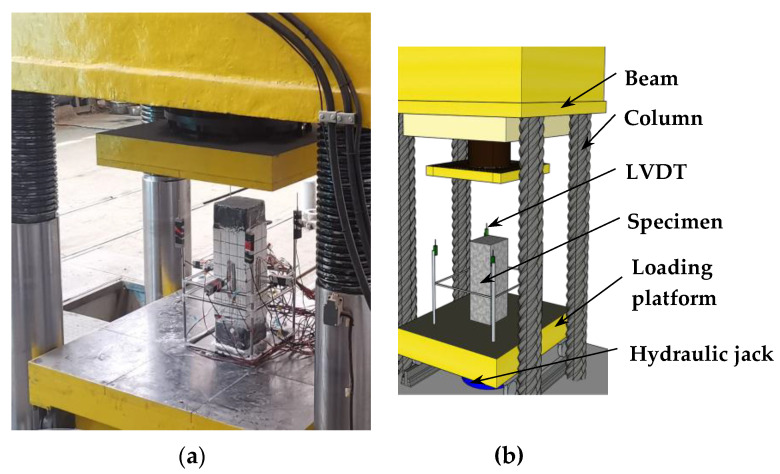
Test setup. (**a**) Diagram of test device. (**b**) Device diagram.

**Figure 7:**
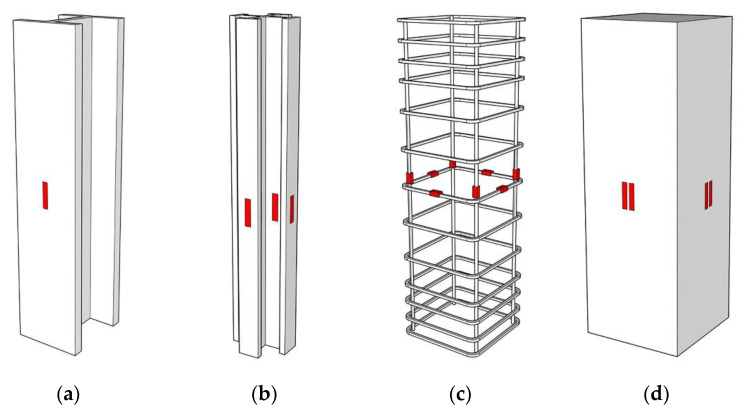
Measuring instrument layout. (**a**) Specimens with H-section steel. (**b**) Specimens with cross-section steel. (**c**) Reinforcement framework, (**d**) Concrete.

The test loading was carried out by the electro-hydraulic servo testing machine (Dongce Testing Machine Technology Co., Ltd., Jinan, China), and the loading device is shown in Figure 6. The test was carried out in a graded loading method. Before the formal load, to ensure the normal operation of the test instrument, a load of 100 kN was applied in advance. At the beginning of the formal test, the load was carried out at a rate of 200 kN/min until the load was 40% of the estimated ultimate load, then the loading rate was reduced to 100 kN/min. When the loading reached 80% of the estimated ultimate bearing capacity, the load method was switched to displacement control, and the loading rate was 0.2 mm/min. After loading to the peak load, the test was ended when the bearing capacity dropped to ~75% of the ultimate load. To facilitate the monitoring of the component forces, strain gauges were pasted on the surface of the section of steel, steel bars, and concrete, and the measuring points are shown in Figure 7. The vertical displacement of the specimen was recorded by the displacement meter in the loading device.

## 3. Test Results and Analysis

### 3.1. Failure Phenomenon

Due to the different design parameters of the specimens, the damage phenomena were different during the stress process. The failure phenomenon of each specimen is described according to the yield state and the changing trend of the bearing capacity of the section steel.

The failure modes of specimens equipped with Q460 (A1~A4) and Q235 steel (A9~A12) are similar, and the failure characteristics are as follows.

**Figure 8:**
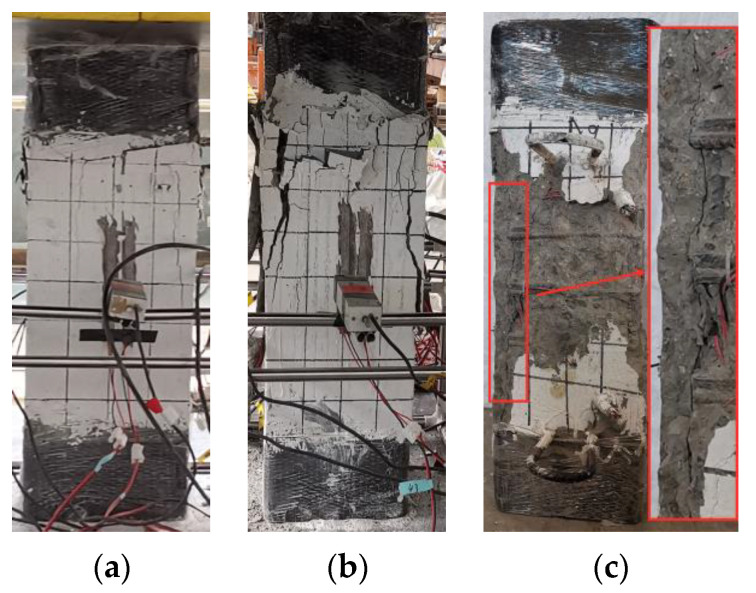
Typical failure modes in each stage of A9. (**a**) Micro-cracks, (**b**) ultimate load, (**c**) stripping of concrete cover.

Figure 8 shows the typical failure characteristics of the specimen. At the initial stage of loading, the specimen was in a linear elastic state, and no cracks appeared on the concrete surface. As shown in Figure 8a, when the load increased to 0.8~0.9 *N*_u_ (*N*_u_ is the ultimate bearing capacity of the specimen), micro-cracks appeared on the side of the upper end of the column; as the load continued to increase, the cracks continued to extend downwards, and the longitudinal bars and the section steel yielded successively. When the specimen equipped with Q235 steel was in a compression state, the section steel yielded before the longitudinal reinforcement. When the strength grade of the section steel was Q460, the longitudinal reinforcement of the composite short column yielded before the section steel. When the specimen reached the ultimate bearing capacity *N*_u_, crack length and crack width developed rapidly. As shown in Figure 8b, the protective concrete layer in the middle section of the specimen cracked and fell off. With the increase of the load, as the concrete at the middle edge of the specimen was crushed, the protective layer continuously fell off, the load-bearing capacity of the specimen was rapidly reduced, and the longitudinal reinforcement bulged outward. Simultaneously, stirrup yielded; as shown in Figure 8c, the concrete of the protective layer was separated from the concrete in the confinement area of the stirrups. Then the rate of reduction of the bearing capacity slowed down until it reached 75% of the ultimate bearing capacity. After the test was over, the broken concrete on the surface of the specimens was removed, and it can be observed that the concrete in the constrained area did not show obvious collapse.

Compared with A1~A4 and A9~A12, the cracks of specimens A5 and A6 (equipped with 3.63% and 5.13% Q690 steel, respectively) appeared later, and the failure process has the following characteristics.

**Figure 9:**
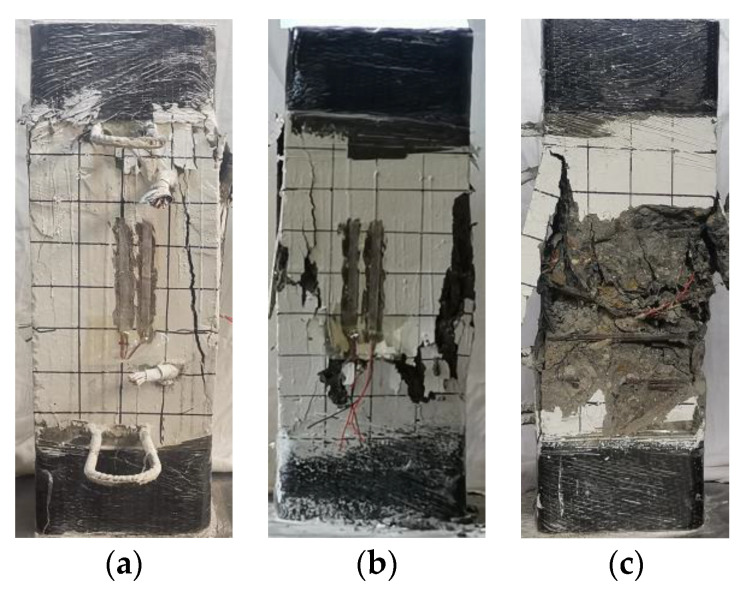
Comparison of cracking degree of concrete. (**a**) A9, (**b**) A5, (**c**) A7.

**Figure 10:**
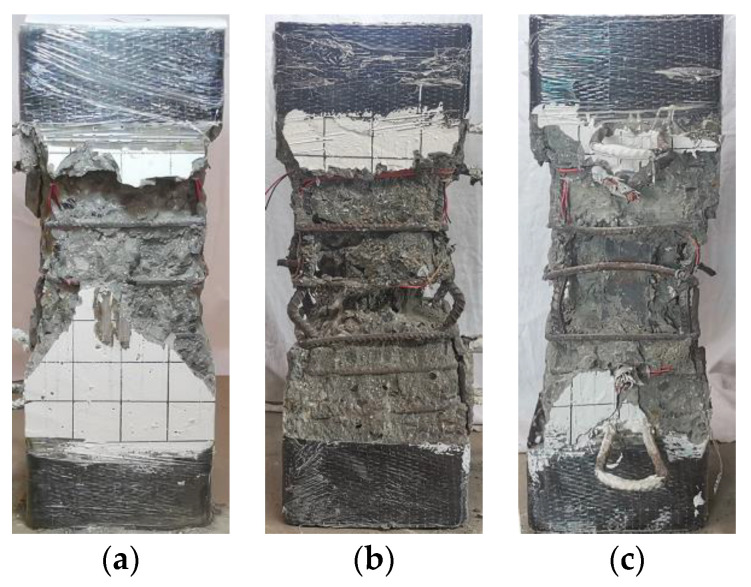
Concrete crushing modes of specimens. (**a**) A9, (**b**) A5, (**c**) A7.

When specimens A5 and A6 reached the ultimate bearing capacity *N*_u_, multiple vertical cracks quickly appeared on the surface of the column, and they developed continuously with the increase of the vertical displacement. At this time, the longitudinal bars yielded, but the section steel had not reached the yielding state. When the bearing capacity dropped to approximately 85% of the peak load, the bearing capacity decreased slowly. When the bearing capacity dropped to ~85% of the peak load, the bearing capacity dropped slowly. The vertical displacement of the specimen when failed increased due to the increase of the strength level of the section steel, and the degree of damage was intensified. Figure 9 and Figure 10 show the characteristics of the failure morphology of different specimens. Compared with specimens A1~A4 and A9~A12, the concrete cracking degree and the final degree of crushing in the middle section of specimens A5 and A6 were more serious.

The specimens A7 and A8 equipped with Q690 steel and steel ratio of 6.20% were loaded to the ultimate load, and then slowly dropped to 0.85 *N*_u_. At this time, the concrete protective layer fell off, the load-displacement curve reached an inflection point, the bearing capacity of the specimen stopped decreasing, and a slow secondary lifting phenomenon occurred. During this process, the Q690 steel yielded, and the surface of the specimen was seriously damaged. When the vertical displacement reached 20~23 mm, accompanied by a loud noise, the specimen lost all bearing capacity, and the test ended. After removing the broken concrete on the surface, it was found that the longitudinal bars buckled outwards, and the stirrups broke.

### 3.2. Load-Displacement Curve

Due to the different design parameters of the specimens, the damage phenomena were different during the stress process. The failure phenomenon of each specimen is described according to the yield state and the changing trend of the bearing capacity of the section steel.

**Figure 11:**
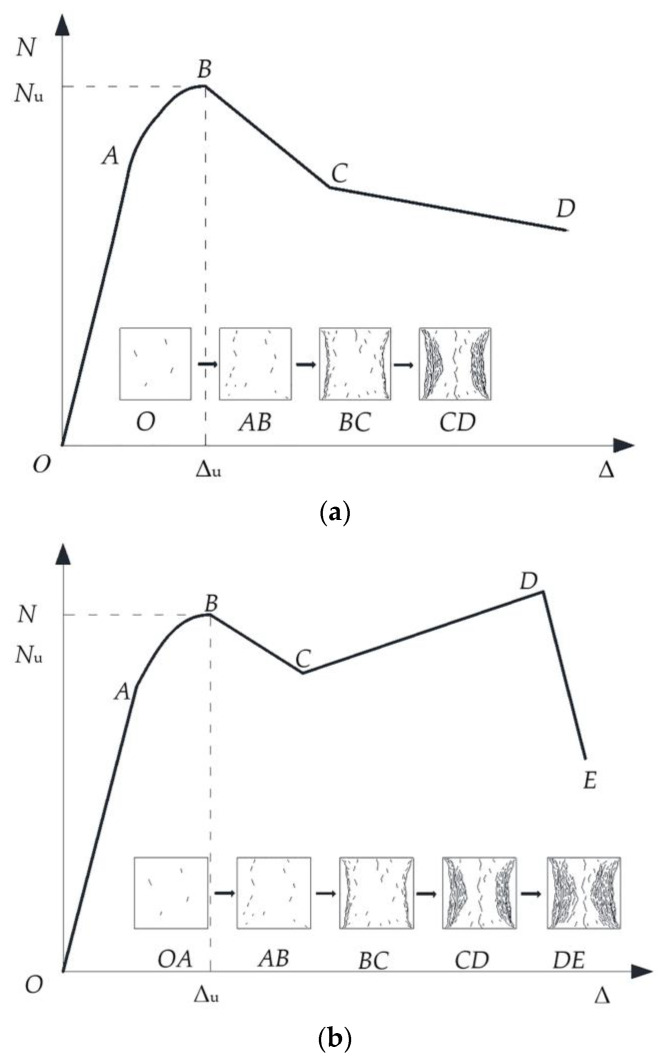
Two ideal specimens *N*-Δ curves. (**a**) Ideal *N*-Δ curve—class Ⅰ. (**b**) Ideal *N*-Δ curve—class Ⅱ.

Figure 11a shows the load-displacement curve of type I. The specimens conforming to the characteristics of this type of curve are A1~A6 and A9~A12. The OA stage is the elastic deformation stage, at which the specimens have linear deformation. At the AB stage, the transverse deformation of each part gradually increases, and the restraint effect of the section steel and stirrups on the concrete in the constrained area continues to increase until the specimen enters the full-section plastic state and reaches the limit bearing capacity. Because the concrete of the protective layer is not restrained, the deformation ability of the specimens is relatively weak at the BC stage. At this stage, it cracks and falls off, and the bearing capacity of the specimen decreases rapidly until the specimen is transformed from the overall axial force to the constrained area of the steel and stirrups. The concrete bears the axial force. At the CD stage, the load is mainly borne by the concrete inside the confinement area of the steel section and stirrups. The concrete in the constrained area of stirrups is jointly restrained by stirrups and steel sections, its deformation ability is improved, its strength degrades slowly, and it can continue to bear the load, so the rate of decrease of the bearing capacity of the specimen is slowed down.

**Figure 12:**
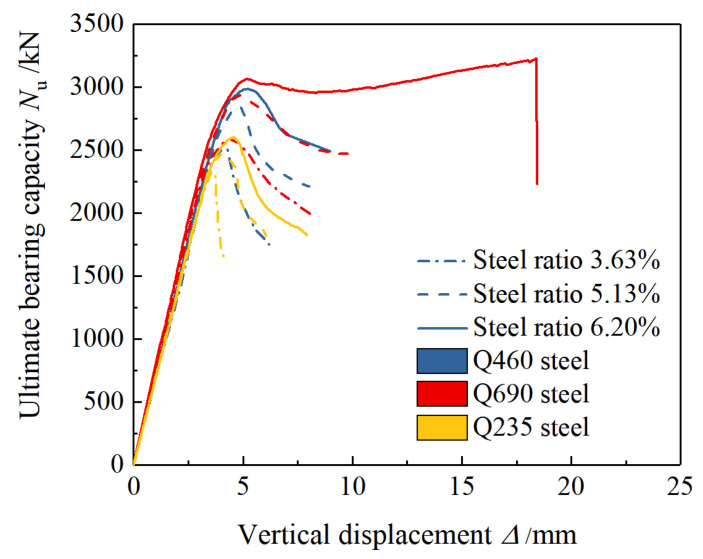
Influence of strength grade and content of steel on load-displacement curves.

**Figure 13:**
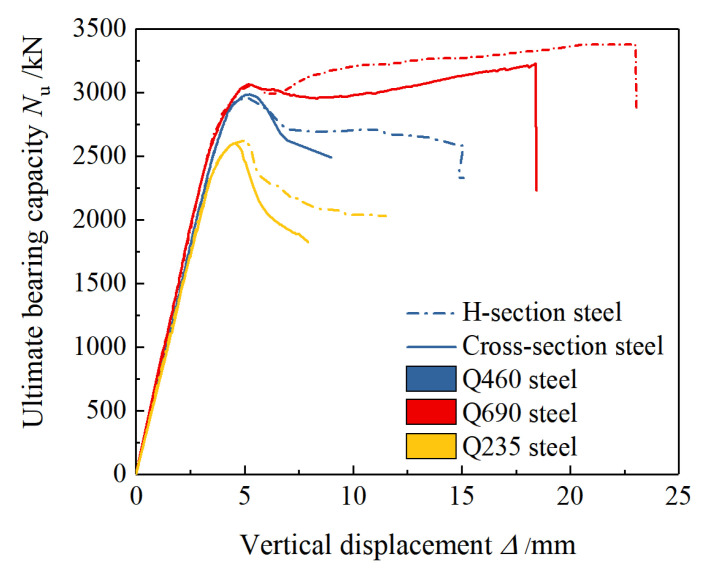
Influence of section form of steel on load-displacement curves.

As shown in Figure 11b, the load-displacement curves of the specimens A7 and A8 (equipped with 6.20% Q690 steel in H-section and cross-section form) both showed a secondary increase. The phenomenon is in line with the characteristics of the type Ⅱ load-displacement curve. At the CD stage, the concrete protective layer has completely failed, but the strength of the concrete in the confinement area of the stirrups degrades slowly. As the sectioned steel reaches the yielding state and enters the strengthening stage, the overall load-bearing capacity of the specimen reaches the lowest point C of the descending section, the displacement continues to increase, the built-in sectioned steel yields and begins to strengthen under the action of axial pressure, and the load changes from falling to rising. At the DE stage, when the displacement increases to a certain extent, the class II curve breaks due to the yield of the stirrup, and the concrete in the confinement area of the stirrup loss of restraint and sudden crushing results in loss of bearing capacity of the specimen. Figure 12 and Figure 13 show the load-displacement curves of the specimens with different steel grades, steel ratios, and section forms of steel.

### 3.3. Ultimate Bearing Capacity

The ultimate bearing capacity of the specimen is shown in Table 4. Compared with the specimens equipped with Q235 steel, when the steel grades were Q460 and Q690, the maximum increase in the bearing capacity of the specimens was 15.3% and 19.0%, respectively. Compared with the specimens equipped with Q460 steel, the bearing capacity of the specimens equipped with Q690 steel increased slightly because the yield strain of the Q690 steel was much larger than the peak compressive strain of the concrete, so the Q690 steel could not yield when the specimens reached the ultimate bearing capacity; therefore the material performances of the high-strength steel were not fully utilized. For the specimens with the same strength grade built-in steel, compared with the specimens with a steel ratio of 5.13%, the highest increases in bearing capacity of the specimens with a steel ratio of 6.20% were 13.6% and 18.8%. The growth rate was relatively large.

### 3.4. Ductility Coefficient

**Figure 14:**
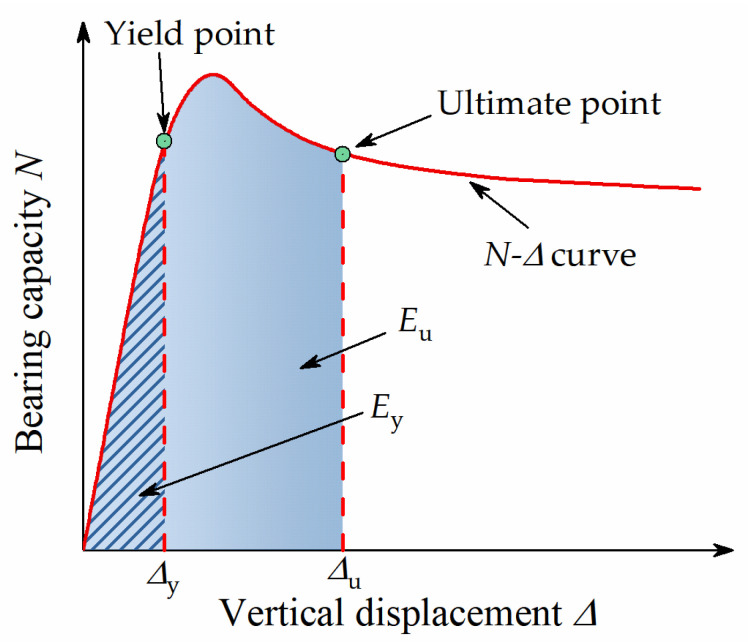
Calculating diagram of ductility factor.

**Figure 15:**
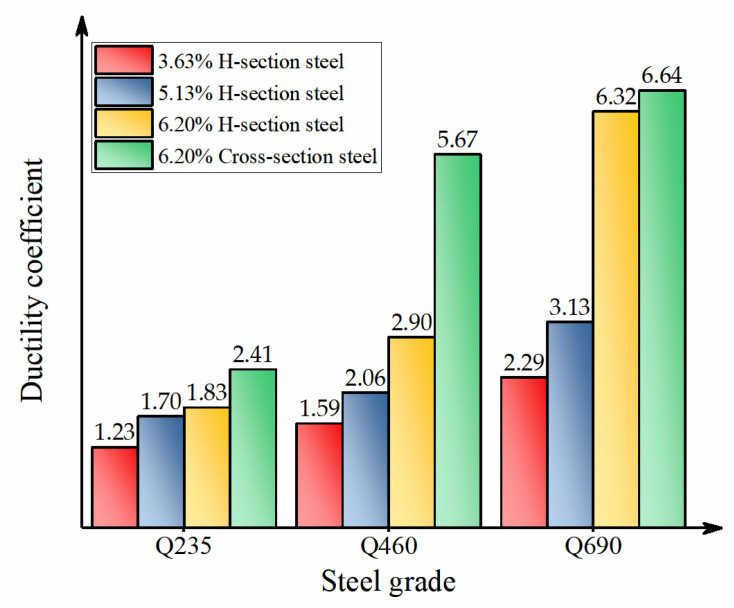
Comparison of ductility of all specimens.

In order to quantitatively analyze the axial ductility of the specimens, the ductility coefficient *μ* is now introduced [45,46], as shown in Equation (1).

(1) $$\mu =E_{u}/E_{y}$$

where, *E*_u_ is the dissipated energy at the ultimate point; *E*_y_ is the dissipated energy at the yield point, as shown in Figure 14. In this study, the ultimate point was taken as the bearing capacity drops to 85% of *N*_u_. Since the *N*-Δ curves of A7 and A8 cannot drop to 85% of *N*_u_, and the phenomenon of the secondary rise occurred, the ultimate points of A7 and A8 were determined from the perspective of deformation. Therefore, the point with three times the deformation of the peak load was selected as the ultimate point. The yield points of the specimens were determined by the energy equivalence method. The calculation results are shown in Table 5, and the ductility comparison of each specimen is shown in Figure 15.

By comparing the calculation results of the ductility coefficient of different specimens, it can be found that the increases of the steel grade and steel ratio had obvious effects on the ductility coefficient of the specimens. When the steel ratio was higher, the increase in the strength grade had a significant impact on the ductility coefficient of the specimens. When the section steel had higher strength, the increase in the steel ratio had a significant impact on the ductility coefficient. When the steel ratio was 6.20%, compared with the specimens equipped with Q235 steel, the ductility coefficients of the specimens equipped with Q460 and Q690 steel were increased by 58.5% and 245.4%, respectively. When the steel grade was Q690, compared with the specimen with a 3.63% steel ratio, the ductility coefficients of the specimens equipped with steel ratios of 5.13% and 6.20% were increased by 36.7% and 176.0%, respectively.

When the form of the steel changes from H-section to cross-section, the ductility coefficient of the specimen equipped with Q235 steel increased by 31.20%, the ductility coefficient of the specimen equipped with Q460 steel increased by 95.20%, and the ductility coefficient of the specimen equipped with Q690 steel increased by 5.1%. When the steel grade was Q235, the displacement difference between the two different cross-section specimens was small when the bearing capacity of the specimen was reduced to about 85% *N*_u_, so the ductility coefficient did not change significantly. However, it can be observed from Figure 11 that when the bearing capacity was reduced to about 75% *N*_u_, the displacement of the specimen equipped with cross-section steel was much greater than that of the specimen equipped with H-section steel. When the steel grade was Q460, the cross-section steel fully exerted the restraint effect on the concrete when the member was stressed, and the ductility coefficient increased significantly. When the steel grade was Q690, the ductility of the specimens equipped with H-section and cross-section steel were both very excellent. Compared with the specimen equipped with H-section steel, the second increasing stage of the load-displacement curves of specimens equipped with cross-section steel showed that the residual bearing capacity was higher, and the ultimate displacement of the specimen was greater.

## 4. Calculation and Analysis of Bearing Capacity

American code AISC360-16, European code EN1994-1-1:2004, and Chinese code JGJ138-2016 all propose the formulas for calculating the axial compressive bearing capacity of SRC columns, but none of them give the calculation formula for the SRC column with high-strength steel. Combined with the test results, the applicability of the above-mentioned formulas for calculating the axial compressive bearing capacity of the specifications for the high-strength SRC column was verified.

### 4.1. The Calculation Method of Current Specifications

The American “Code for Design of Steel Structure Buildings” (AISC360-16) adopts the method equivalent to the section steel of the outer reinforced concrete part and calculates according to the steel structure design method. The calculation formula of the axial compression is:

(2) $$P_{n}=\left\{ \begin{array}{ll} P_{n0}\left(0.658^{\frac{P_{n0}}{P_{e}}}\right) & \frac{P_{n0}}{P_{e}}\leq 2.25\\ 0.877P_{e} & \frac{P_{n0}}{P_{e}}>2.25 \end{array}\right.$$

(3) $$P_{n0}=F_{y}A_{s}+F_{\mathrm{ysr}}A_{\mathrm{sr}}+0.85f_{c}^{\prime }A_{c}$$

(4) $$P_{e}=\pi^{2}(EI_{\mathrm{eff}})/L_{c}^{2}$$

where, *A*_s_, *A*_sr_, *A*_c_ represent the section area of section steel, steel bar, and concrete, respectively; *F*_y_, *F*_ysr_, *f*_c_′ represent the compressive strength of section steel, steel bar, and concrete, respectively; *EI*_eff_ represents the effective stiffness of the section; *L*_c_ represents the effective length of the member.

For biaxially symmetric steel-concrete composite columns, the calculation formula for the axial compressive bearing capacity of the European “Code for Design of Steel and Concrete Composite Structures” (EN1994-1-1:2004) is as follows:

(5) $$N_{\mathrm{Ed}}\leq \chi N_{\mathrm{pl},\mathrm{Rd}}$$

(6) $$N_{\mathrm{pl},\mathrm{Rd}}=A_{a}f_{\mathrm{yd}}+0.85A_{c}f_{\mathrm{cd}}+A_{s}f_{\mathrm{sd}}$$

where, *A*_a_, *A*_c_, *A*_s_, represent the cross-sectional area of section steel, concrete, and reinforcement, respectively; *f*_yd_, *f*_cd_, *f*_sd_ represent the compressive strength of section steel, concrete, and reinforcement, respectively; *χ* represents the buckling reduction coefficient considering the relative slenderness ratio, confirmed according to the European “Code for Design of Steel Structures” (EN 1993-1-1:2005) [47].

The calculation formula for the axial compressive bearing capacity of steel-concrete composite columns from “Code for Design of Composite Structures” (JGJ 138-2016) is:

(7) $$N\leq 0.9\varphi (f_{c}A_{c}+f_{y}^{\prime }A_{s}^{\prime }+f_{a}^{\prime }A_{a}^{\prime })$$

where, *f*_c_, *f*_y_′, *f*_a_′ represent the design value of compressive strength of concrete, steel bar, and section steel, respectively; *A*_c_, *A*_s_′, *A*_a_′ represent the cross-sectional area of concrete, steel bar, and section steel, respectively; *φ* represents the axial compression stability coefficient, which can be obtained according to the slenderness ratio look-up table.

### 4.2. Comparison of Test Results and Calculation Results

**Figure 16:**
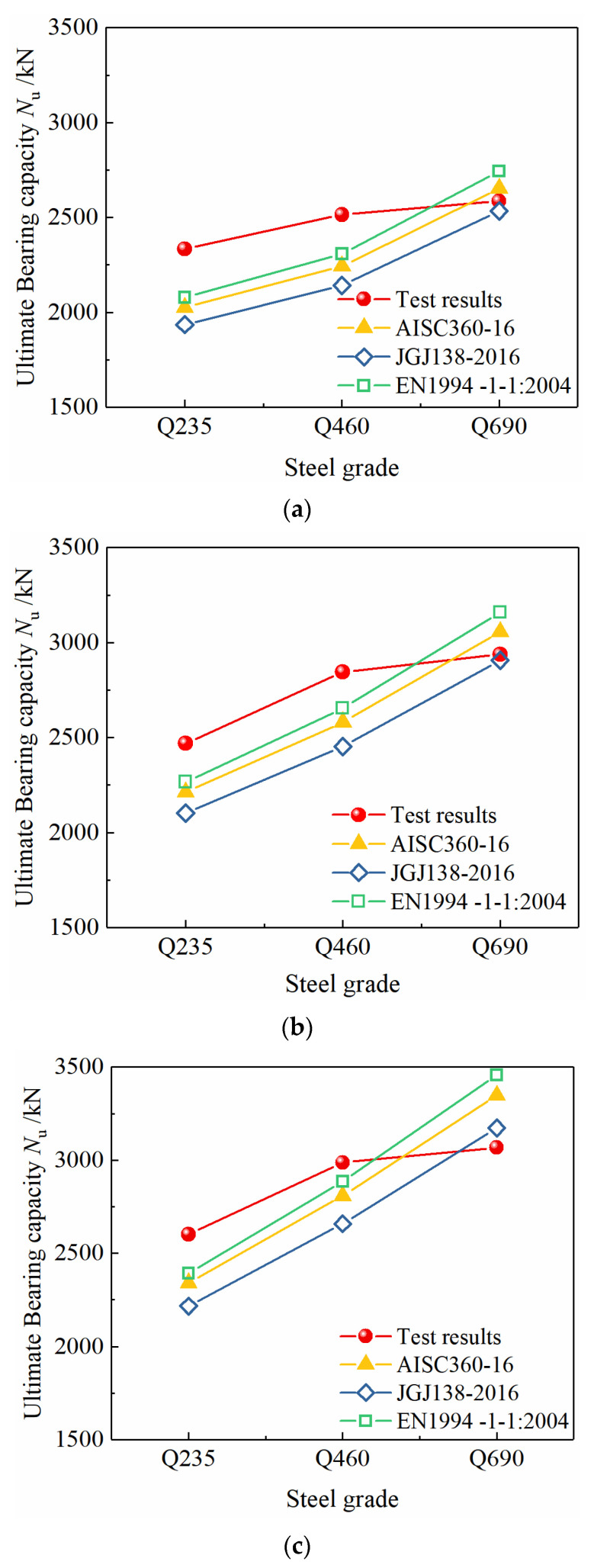
Effect of steel grade on bearing capacity of specimens. (**a**) Steel ratio 3.63%, (**b**) steel ratio 5.13%, (**c**) steel ratio 6.20%.

As shown in Figure 16, when the steel content is constant, as the strength grade of the section steel increases from Q235 to Q460, the bearing capacity of the specimens is significantly improved. Based on the different specifications, the calculation results are much smaller than the test results, the norms of all countries are conservative, and the changing trend of the calculated value of the bearing capacity is consistent with the test value. The European standard calculation results are the closest to the test results, and the Chinese standard calculation results are the most conservative. When the existing standard calculation methods are applied to the calculation of the axial compressive bearing capacity of the Q690 high-strength steel-concrete composite specimens, the calculation results can not predict the bearing capacity of the columns accurately, because compared to Q235 and Q460 steel, the yield strain of Q690 steel is much larger. Because of the limitation of the compressive deformation capacity of concrete, the specimens reach the ultimate bearing capacity when the concrete is crushed, but the Q690 steel has not yet yielded, so the strength has not been fully developed. If the yield strength of Q690 section steel is still used to calculate the bearing capacity based on the strength superposition theory, the contribution of the high-strength section steel to the axial load bearing capacity would be overestimated, resulting in unsafe calculation results. Therefore, it is not appropriate to directly use the existing calculation method for the calculation of the bearing capacity of the Q690 high-strength steel-concrete composite column specimens.

**Figure 17:**
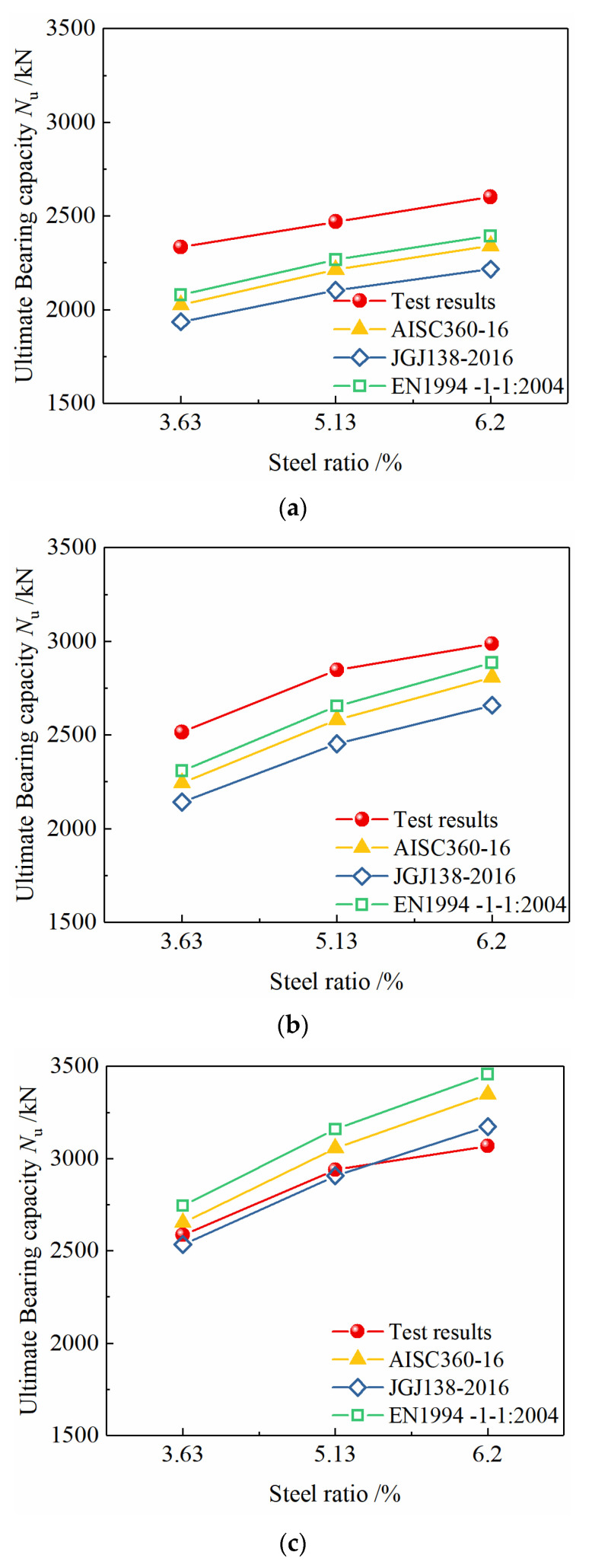
Effect of steel ratio on bearing capacity of specimens. (**a**) Steel grade Q235, (**b**) steel grade Q460, (**c**) steel grade Q690.

As shown in Figure 17, when the strength grade of the section steel is constant, as the steel content increases from 3.63% to 5.13% and 6.20%, the bearing capacity of the specimen increases significantly, and the test results and the standard calculation results have the same change trend.

### 4.3. Design Suggestion

**Figure 18:**
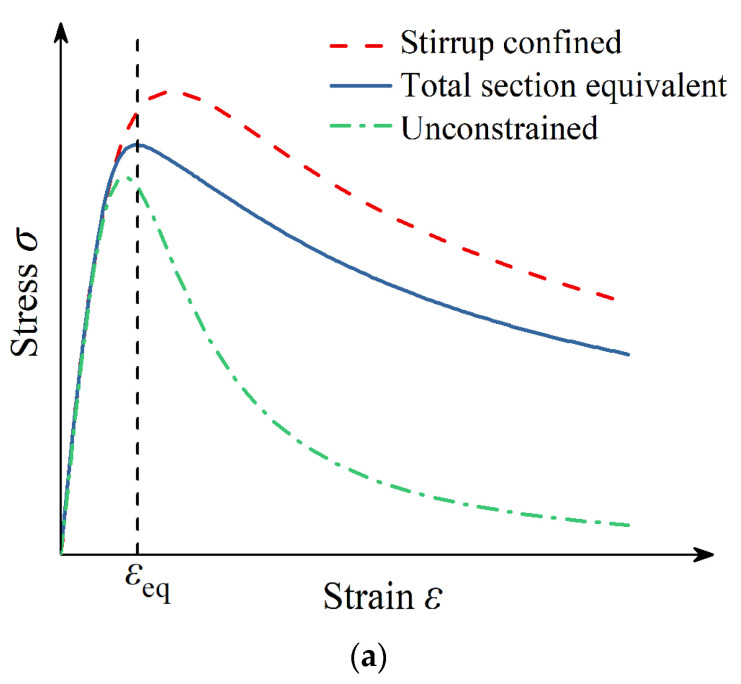

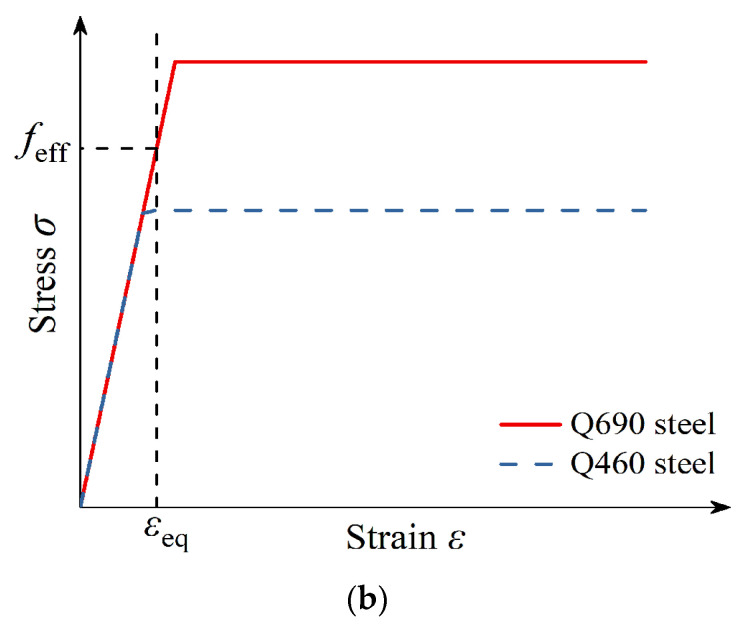
Determination of effective compressive strength of high-strength steel. (**a**) Equivalent total section concrete peak strain. (**b**) Effective compressive strength of high-strength steel.

**Figure 19:**
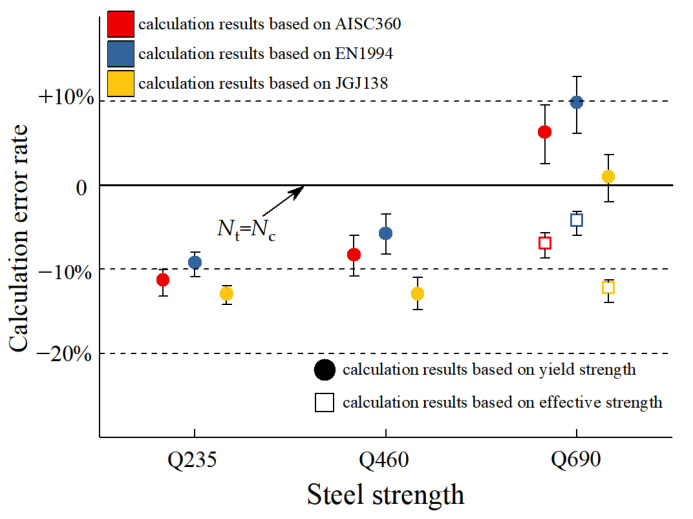
Influence of strength limit of steel on calculation results. *N*_c_ and *N*_t_ represent the calculation results of the bearing capacity and the test bearing capacity. Color (red, blue, and yellow) is used to distinguish different standards. Shapes (solid circles and hollow squares) used to distinguish steel strength values.

According to the above test results and theoretical analysis, Q690 steel can greatly improve the residual bearing capacity and deformation capacity of the specimens. However, because the specimens reach the ultimate bearing capacity and the high-strength section steel fails to yield, it is not advisable to use the existing bearing capacity calculation formulas when designing this type of member. A comprehensive consideration of the strength degradation of the protective layer of concrete and the improvement of the strength and deformation performance of the concrete in the confinement area of the stirrups, if no measures are taken to ensure the compressive deformation capacity of concrete, when calculating the axial compressive bearing capacity of high-strength steel-concrete composite short columns, the strength of the section steel should be taken from its effective compressive strength:

(8) $$f_{\mathrm{eff}}=E_{s}\varepsilon_{\mathrm{eq}}$$

where, *E*s is the elastic modulus of section steel, as shown in Figure 18. According to the weight of the unconfined concrete and the concrete in the constrained area in the full-section concrete, the equivalent full-section concrete stress-strain curve has been calculated, as shown in Figure 18a. The strain value corresponding to its peak point is the equivalent full-section concrete peak strain *ε*_eq_, taking the strain value *ε*_eq_ on the stress-strain curve of the high-strength section of steel as the corresponding stress is the effective compressive strength *f*_eff_ provided by the high-strength section steel, as shown in Figure 18b. The influence of this method on the calculation results of bearing capacity is shown in Figure 19.

## 5. Finite Element Analysis

### 5.1. Model Establishment

In the failure process of the specimens equipped with Q690 steel, the compressive strength of the steel is not fully utilized, resulting in insufficient overall bearing capacity of the specimen. When the steel reaches the yield stress value, the protective layer concrete has severely cracked or even fell off, which seriously affects the applicability of the structure. To meet the requirements of engineering application, it is necessary to propose a measure to improve the concrete deformation capacity of the component. To solve this problem, steel fiber concrete can be applied to high-strength SRC columns.

Studies have shown that the incorporation of steel fibers can effectively improve the deformability of concrete [48,49]. The numerical analysis of the representative specimens A3, A4, A7, and A8 in the test was carried out through the ABAQUS finite element analysis software. Based on the research [50], the proposed outsourcing hexahedron method uses python scripts to insert randomly distributed steel fibers inside the specimen. By comparing the stress characteristics of the specimen before and after inserting the steel fiber, the influence of the incorporation of steel fibers to the bearing capacity and the force behavior were analyzed for the specimen.

**Figure 20:**
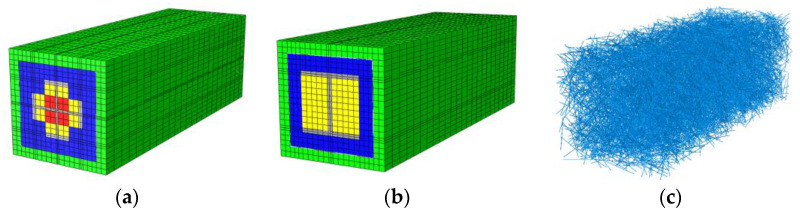
Finite element models and the meshing plan. (**a**) Equipped with cross-section steel model. (**b**) Equipped with H-section model. (**c**) Steel fiber model.

In the model, the C3D8R hexahedral reduced integral element was selected for concrete and section steel element type, and T3D2 three-dimensional truss element was selected for reinforcement and steel fiber element type. The friction was defined at the interface between the concrete and the steel to consider the bond. The model grid was divided with the size ratio proposed by Ehobody et al. [51]. After convergence analysis, we selected the grid division scheme, as shown in Figure 20, and imposed constraints based on the actual loading device.

### 5.2. Definition of Material Properties

The compressive capacity and deformation capacity of concrete can be significantly improved under the lateral restraint. Scholars have proposed a variety of constitutive curves of concrete under restraint. Among them, the constitutive relationship of restrained concrete proposed by Mander et al. [52] considered factors, such as the stirrup ratio, stirrup strength, stirrup form, and the concrete compressive strength; the expression is as follows:

(9) $$\sigma =\frac{f_{\mathrm{cc}}xr}{r-1+x^{r}}$$

where *σ* is the concrete stress; *f*_cc_ is the compressive strength of concrete considering the lateral restraint effect; *x* = *ε*/*ε*_cc_; *ε* is the concrete strain *E*_sec_ = *f*_cc_/*ε*_cc_, *f*_cc_ = *kf*_c0_, *ε*_cc_ = [1 + 5(*k* − 1)]*ε*_c0_, *E*_c_, *E*_sec_ are the elastic modulus and secant modulus of concrete; *f*_c__0_ is the axial compressive strength of concrete; *k* is the strength improvement coefficient of constrained concrete.

**Figure 21:**
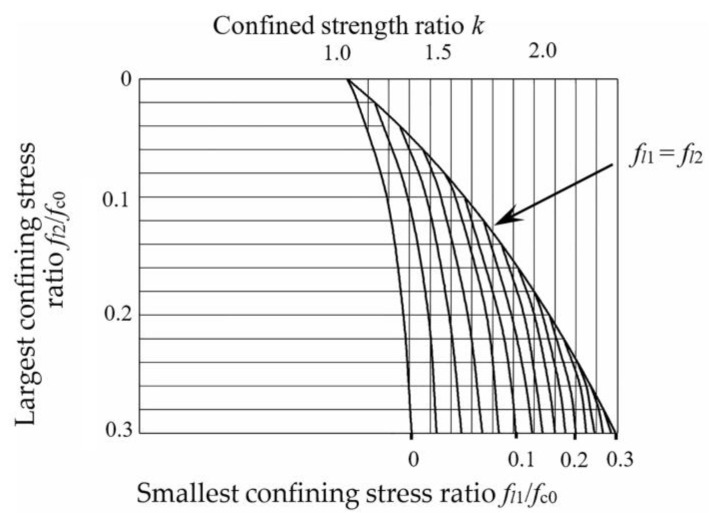
Triaxial diagram of confined concrete strength improvement coefficient.

For unconstrained concrete, *k* = 0; for concrete in the area confined by stirrups, Mander gives the calculation method as:

(10) $$k=-1.254+2.254\sqrt{1+7.94\frac{f_{l}^{\prime }}{f_{c0}}}-2\frac{f_{l}^{\prime }}{f_{c0}}$$

where *f*_c__0_ is the axial compressive strength of concrete and *f_l_*′ is the effective lateral restraint stress [53] for the concrete in the effective confinement area of section steel and stirrups. Combined with the calculation method of effective restraint stress of section steel given by Zhao et al. [54] and the theory of linear superposition of restraint of joint restraint area proposed by Feng et al. [55], the triaxial figure proposed by Mander can be used to determine the strength improvement coefficient k, as shown in Figure 21.

The material performances of sectioned steel and reinforcing steel adopt an ideal elastoplastic constitutive model, and its mechanical performance index was adopted according to the results of the material property test. As shown in Table 6, combined with related research and design specifications, the simulation parameters of steel fiber were determined.

### 5.3. Analysis Result

**Figure 22:**
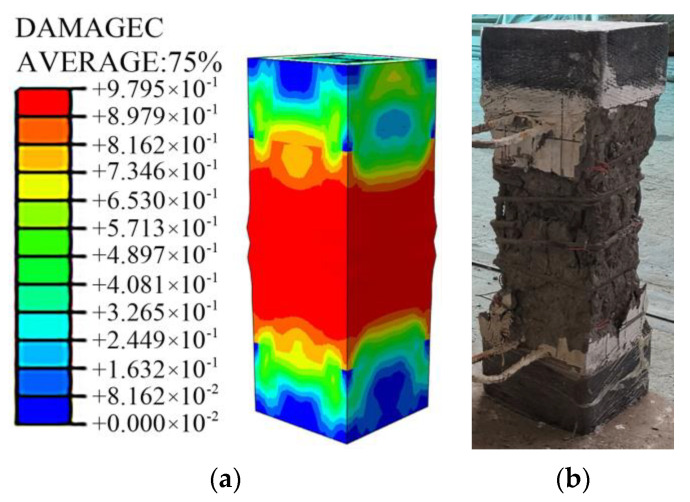
Comparison between simulated failure modes and test results. (**a**) Simulated failure mode, (**b**) test result.

**Figure 23:**
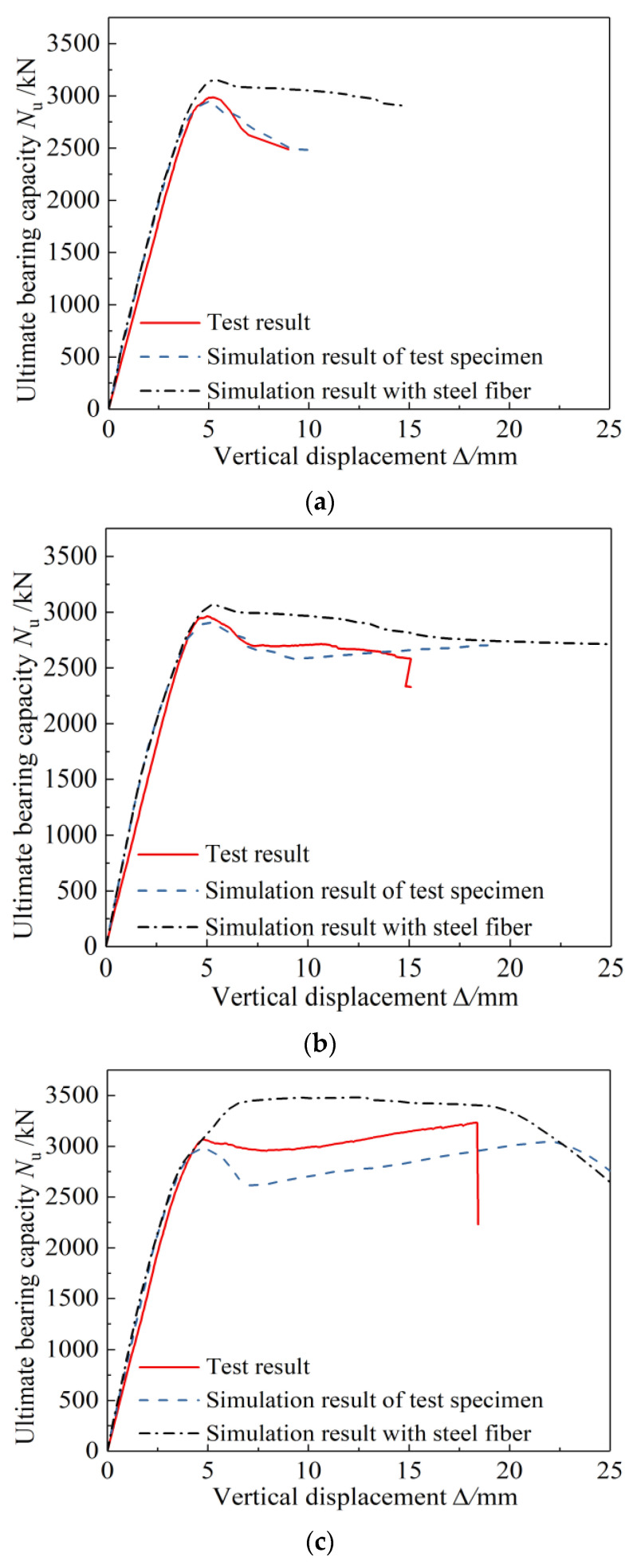

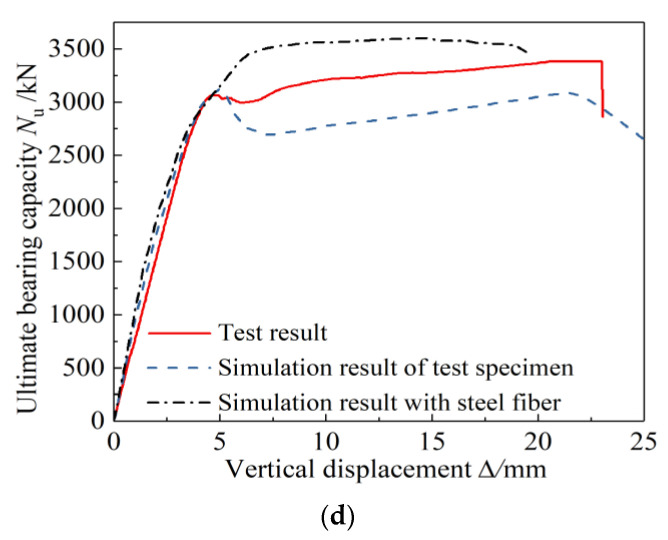
Comparison of load-displacement curves between two simulation results with test results. (**a**)A3, (**b**) A4, (**c**) A7, (**d**) A8.

As shown in Figure 22, the damage pattern of the simulation analysis was in good agreement with the test results. Figure 23 shows the test results of typical specimens and the load-displacement curves of the simulation results before and after the concrete was added with steel fiber. The simulation curve without steel fiber was in good agreement with the test result curve, indicating that the model can simulate and analyze the stress process of this type of specimen accurately; the reinforcement of fibers had little effect on the ultimate bearing capacity of specimens equipped with Q460 steel. However, it can significantly enhance the ultimate bearing capacity of the specimens equipped with Q690 steel so that the strength can be fully exerted. Compared with the simulation results of the specimens without steel fibers, the simulation results with steel fibers of the bearing capacity of specimens A7 and A8 increased by 15.5% and 13.9%, respectively.

**Figure 24:**
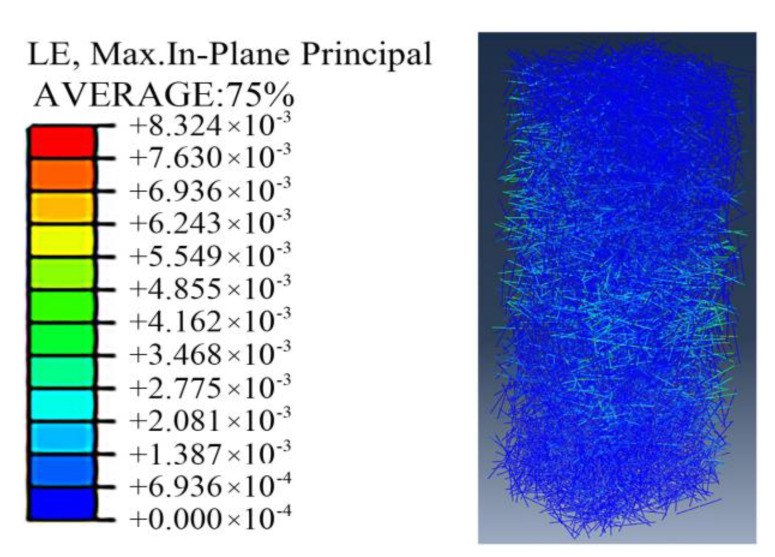
Simulation results of steel fiber strain.

**Figure 25:**
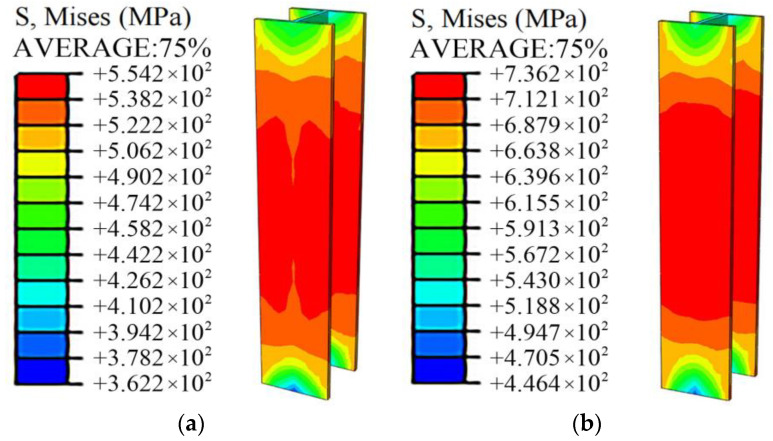
Effect of steel fiber reinforcement on the stress of Q690 steel. (**a**) Without steel fiber, (**b**) incorporation of steel fiber.

As shown in Figure 24, in the process of concrete compression, the steel fibers distributed along the cross-section of the specimen at the place where the concrete stress is relatively large produced obvious tensile strain. As shown in Figure 25, by comparing and analyzing the stress cloud diagram of section steel, it can be observed that after the concrete is reinforced by steel fiber, when the specimen reaches the ultimate bearing capacity, Q690 steel reaches the yield state, which can give full play to the strength of the material. The passive restraint measure provides a lateral restraint effect for the concrete, which can enhance the deformability of the full-section concrete to match the Q690 high-strength steel.

At present, scholars have conducted research on the deformation performance of steel fiber concrete [56,57,58,59,60]. Based on the analysis of the test results, the conditions that the high-strength steel is applied to the SRC columns are put forward: *f*_cc_ ≥ *f*_y_/*E*_s_. To meet this condition, the peak strain of concrete can be increased by adding steel fiber so that it is not less than the yield strain of sectioned steel to ensure that the mechanical performance of high-strength sectioned steel can be fully exerted.

## 6. Conclusions

In this paper, the axial load test, theoretical analysis, and ABAQUS simulation of eight HSSRC columns equipped with Q460 and Q690 steel and four SRC columns equipped with Q235 steel were carried out. The conclusions are as follows:

(1) For the specimens with the same steel ratio, the bearing capacity and ductility of SRC columns increase with the increase of steel strength. Compared with the specimens with Q235 steel, the bearing capacity and the ductility of the specimens equipped with Q460 steel increased by 7.8–15.3%, 21.2–135.3%, respectively; the bearing capacity and the ductility of the specimens equipped with Q690 steel increased by 13.2–24.1%, 84.1–245.4%, respectively.
(2) For the specimens with the same strength grade, the bearing capacity and ductility of SRC columns can be significantly improved by increasing the steel ratio. Compared with the specimens equipped with a 3.63% steel ratio, the bearing capacity and ductility of the specimens equipped with 5.13% steel ratio increased by 5.8–13.6%, 29.6–38.2%, respectively; the bearing capacity and the ductility of the specimens equipped with a 6.20% steel ratio increased by 11.5–18.6%, 48.8–176.0%, respectively.
(3) For the specimens with the same steel ratio, when the form of steel section is transformed from H-section to cross-section, in terms of bearing capacity, the bearing capacity of SRC columns did not change significantly; in terms of ductility, when the steel grade was Q460, the ductility of the specimen equipped with cross-section steel was 95.2% higher than the ductility of the specimen equipped with H section. When the steel grade was Q690, the ductility of the specimens equipped with H-section and cross-section steel were both very excellent.
(4) Comparing the test results with the calculation results of the existing approach, it is found that the existing calculation approach can accurately calculate the axial bearing capacity of SRC columns equipped with Q460 steel, but it is unable to calculate the axial bearing capacity of SRC columns equipped with Q690 steel. Therefore, the method of determining the effective compressive strength of high-strength steel in SRC columns is proposed. The results show that this method can accurately calculate the axial bearing capacity of HSSRC columns and apply the calculation results to engineering applications.
(5) The simulation results show that the bearing capacity of HSSRC columns with added steel fibers is higher than the HSSRC columns without steel fibers. Therefore, steel fibers are suggested to be incorporated into the HSSRC columns.

## 7. Research Limitation

In this paper, the axial load test of HSSRC short columns was carried out, and the conclusions were obtained by combining theoretical analysis. However, the research still has limitations:

(1) The parameters studied in this paper were the influence of the steel grade, the steel ratio, and the section form of the steel. Subsequently, parameters such as slenderness ratio, stirrup form, and concrete strength can be added for the test.
(2) Based on the theoretical analysis, this paper proposed to improve the deformation performance of concrete by steel fibers to make full use of the compression performance of high-strength steel, which is verified by an ABAQUS simulation. The conclusion can be verified by experiments.

## Figures and Tables

**Table 1:** Research status in various countries.

Author	Strength/MPa	Type	Number of Specimen	Method	Main Conclusion
Gu [23]	459–737	HSRC	12	experiment	The predicted values based on Code ACI318-14 for the bearing capacity of such specimens are too conservative.
Li [24]	437–629		9		The effect of high-strength steel bars on the bearing capacity of specimens is not obvious.
Li [25]	437–629		9		High-strength steel bars do not yield. It is suggested that the steel strength should be 500 MPa when calculating the axial capacity of such specimens.
Mohsen [26]	520–670		39		The ACI 318 model over-predicts the capacities for the 10 columns out of 26 columns tested with nominal 2% steel reinforcement ratio.
Wang [27]	522–906	CFHSST	13	experiment	The codes overestimate the bearing capacity of UHPC filled high-strength steel tubular short columns.
Du [28]	724–741		10		It is pointed out that the limitation of superposition theory—the influence of constraints are not considered, which makes the calculation results inaccurate.
Wei [29]	359–1153		15	experiment and simulation	Concrete enters plasticity prior to high-strength steel tube, shear failure is obvious, and constraint of high strength steel tube is insufficient.
Cai [30]	629–1022		34		The predicted value based on Eurocode is generally unconservative.
Fang [31]	760–782		15		The ultimate loads based on the Eurocode 4 and the approach from Zhu and Chan [32] were overestimated by 18–28%.
Ehab [33]	275–690	HSSRC	54	simulation	EC4 overestimated the flexural bearing capacity of eccentric columns.
Wang [34]	276–774		12	experiment	Eurocode 4 and AISC360 are too conservative, and JGJ138 is unconservative at eccentricity 0.6.
Lai [35]	590–646		12		EN 1994-1-1 gives unconservative estimation of the N-M interaction strength curve of CES columns with high-strength steel S500.
Li [36]	439–888		8		Steel contribution ratio is identified as a new parameter for the fire resistance design of CES columns, but it is not mentioned in the Codes.
Li [37]	439–888		5	experiment and simulation	The result of the fire resistance time of CES columns based on Code EN1994-1-2 is unconservative when the applied load on CES column specimens exceeds the limiting value.
Yang [38]	496–530		2		The predicted values based on AISC360 are too conservative, resulting in material waste.
Yang [39]	517		1		The predicted values based on N-M correlation curve method are conservative.

HSRC represents high-strength reinforcement concrete; CFHSST represents concrete filled high strength steel tube; HSSRC represents high-strength steel reinforced concrete.

**Table 2:** Main parameters of specimens.

Specimen Designation	Steel Grade	Steel Ratio to Concrete	Dimensions of Steel (b × h × t1 × t2)	Stirrups Spacing	Section Form of Steel
A1Q4S3-H(A1)	Q460	3.13%	100 × 100 × 5 × 5	70	H
A2Q4S5-H(A2)	Q460	5.13%	100 × 106 × 8 × 5	70	H
A3Q4S6-H(A3)	Q460	6.20%	110 × 106 × 8 × 8	70	H
A4Q4S6-+(A4)	Q460	6.20%	50 × 106 × 8 × 5	70	+
A5Q6S3-H(A5)	Q690	3.13%	100 × 100 × 5 × 5	70	H
A6Q6S5-H(A6)	Q690	5.13%	100 × 106 × 8 × 5	70	H
A7Q6S6-H(A7)	Q690	6.20%	100 × 106 × 8 × 8	70	H
A8Q6S6-+(A8)	Q690	6.20%	50 × 106 × 8 × 5	70	+
A9Q2S3-H(A9)	Q235	3.13%	100 × 100 × 5 × 5	70	H
A10Q2S5-H(A10)	Q235	5.13%	100 × 106 × 8 × 5	70	H
A11Q2S6-H(A11)	Q235	6.20%	110 × 106 × 8 × 8	70	H
A12Q2S6-+(A12)	Q235	6.20%	50 × 106 × 8 × 5	70	+

**Table 3:** Mechanical properties of steel.

Grade	Yield Strength		Ultimate Strength		Elongation Ratio
	*f*_y_/MPa	C_v_	*f*_u_/MPa	C_v_	*δ*/%
Q235	291.0	5.4%	453.1	4.3%	32.3%
Q460	469.1	7.9%	557.3	8.3%	25.0%
Q690	735.1	1.1%	822.3	0.9%	19.2%
HRB400	443.2	4.5%	594.9	3.7%	27.8%

C_v_ represents coefficient of variation.

**Table 4:** Comparison of test results with calculation results calculated codes.

Specimens	Test Results	Simulation Results		Calculation Results								
	Bearing CapacityN_u_/kN	Bearing CapacityN_a_/kN	Simulation Error	AISC360-16			EN1994-1-1:2004			JGJ138-2016		
				N_u_^ca^/kN	Error Rate		N_u_^cc^/kN	Error Rate		N_u_^cj^/kN	Error Rate	
					Single	Average		Single	Average		Single	Average
A1Q4S3-H	2514.0	2457.3	−2.3%	2241.9	12.1%	9.1%	2307.8	8.9%	6.15%	2141.2	17.4%	14.9%
A2Q4S5-H	2845.6	2787.9	−2.0%	2581.2	10.2%		2654.5	7.2%		2452.2	16.0%	
A3Q4S6-H	2987.5	3032.5	1.5%	2807.7	6.4%		2884.7	3.6%		2658.6	12.4%	
A4Q4S6-+	2961.2	3010.4	1.7%	2753.9	7.5%		2822.7	4.9%		2602.8	13.8%	
A5Q6S3-H	2585.6	2635.3	1.9%	2651.6	−2.5%	−5.8%	2744.6	−5.8%	−8.9%	2534.2	2.0%	−0.93%
A6Q6S5-H	2938.5	2988.1	1.7%	3055.4	−3.8%		3159.0	−7.0%		2906.2	1.1%	
A7Q6S6-H	3067.7	2976.7	−3.0%	3346.5	−8.3%		3456.8	−11.3%		3173.5	−3.3%	
A8Q6S6-+	3060.6	3126.5	2.2%	3352.4	−8.7%		3455.3	−11.4%		3172.2	−3.5%	
A9Q2S3-H	2332.9	2327.2	−0.2%	2025.5	15.2%	12.8%	2079.0	12.2%	10.2%	1935.2	20.5%	18.73%
A10Q2S5-H	2468.9	2407.1	−2.5%	2212.5	11.6%		2266.1	9.0%		2102.6	17.4%	
A11Q2S6-H	2601.0	2559.9	−1.6%	2339.8	11.2%		2392.9	8.7%		2216.0	17.4%	
A12Q2S6-+	2623.3	2546.3	−2.9%	2318.8	13.1%		2367.3	10.8%		2193.0	19.6%	

Simulation error = *N*_a_*/N*_u_ *−* 1. Error rate = *N*_u_*/N*_u_^c^* *−* 1*. N*_u_^ca^, *N*_u_^cc^, *N*_u_^cj^ represent the calculation results according to the standards of the United States, Europe, and China, respectively. The details of the simulation method and the simulation verification are shown in Section 5.

**Table 5:** Loads and displacements of characteristic points.

Specimens	Δ_y_/mm	*E*_y_	Δ_u_/mm	*E*_u_	*μ*
A1Q4S3-H	3.70	4660.9	4.85	7415.84	1.59
A2Q4S5-H	3.89	5230.2	5.96	10,790.8	2.06
A3Q4S6-H	4.15	6115.6	8.35	17,757.3	2.90
A4Q4S6-+	4.16	6163.9	15.06	34,940.8	5.67
A5Q6S3-H	3.76	5138.5	6.48	11,783.1	2.29
A6Q6S5-H	3.97	5869.2	8.53	18,357.5	3.13
A7Q6S6-H	4.16	6539.6	14.37	41,312.8	6.32
A8Q6S6-+	4.18	6637.4	13.83	44,051.8	6.64
A9Q2S3-H	3.42	4017.9	3.83	4936.8	1.23
A10Q2S5-H	3.58	4422.0	4.88	7526.1	1.70
A11Q2S6-H	3.84	5009.8	5.51	9190.1	1.83
A12Q2S6-+	3.82	5144.2	6.81	12,382.4.	2.41

Where, Δ_y_ is the displacement corresponding to the yield point, Δ_u_ is the displacement corresponding to the ultimate point.

**Table 6:** Simulation parameters of steel fibers.

Type	*f*_sf_/MPa	*ρ*_f_/%	*d*_f_/mm	*l*_f_/mm	*l*_f_/*d*_f_
Flat	400	1.0	0.8	60	75

*f*_sf_, *ρ*, *d*_f_, *l*_f_ represent the tensile strength, volume content, diameter, and length of steel fiber, respectively.

## Data Availability

The data presented in this study are available on request from the corresponding author.
